# Cataract

**Published:** 1824-09-01

**Authors:** 


### CATARACT.

1. *Lectures on the Operative Surgery of the Eye: being the Substance of that Part of the Author's Course of Lectures on the Principles and Practice of Surgery which relates to the Diseases of that Organ; published for the purpose of assisting in bringing the Management of these Complaints within the Principles which regulate the Practice of Surgery in general.* By G. J. GUTHRIE, Deputy Inspector of Hospitals during the Peninsular War, Surgeon to the Royal Westminster Infirmary for Diseases of the Eye, Consulting Surgeon to the Western Dispensary for the Diseases of Women and Children, Assistant Surgeon to the Westminster Hospital, Lecturer on Surgery, &c. &c. &c.
2. *On the Nature and Symptoms of Cataract, and on the Cure of that Disease in its early Stages, by a Mode of Practice calculated to prevent the Occurrence of Blindness, and to render unnecessary the Operations of Couching and Extraction. Illustrated by Cases.* By JOHN STEVENSON, Esq. Fellow of the Royal College of Surgeons; and Surgeon, Oculist, and Aurist to His Royal Highness the Duke of York, &c. &c. London. Whittaker, 1824.
3. *Practical Observations on the Removal of every Species and Variety of Cataract, by Hyalonyxis or Vitreous Operation; illustrated by Cases, with Critical and General Remarks on the other Methods employed.* By JOHN BOWEN, M.D. Fellow of the Royal College of Physicians, Edinburgh; Member of the Royal College of Surgeons, London; Member of the Fiso-critics of Siena, and of the Arcadia of Rome; Corresponding Member of the Georgofili of Florence, &c. &c. &c. London. Callow, 1824.

WE have already dedicated two articles to the review of Mr. Guthrie's Lectures on the eye, and now resume the subject, with the intention of confining ourselves, on the present occasion, exclusively to the consideration of cataract. The vast importance of the subject requires no proof. For the erroneous and absurd opinions that were once entertained, both upon the nature and treatment of cataract, we must refer to systematic works upon the subject. Our pages may be more profitably occupied by a condensed statement of what we do know, than by an erudite and lengthened detail of what our ancestors did not know. Briefly, however, we may remark that, in 1709, Heister, St. Yves, Petit, and Daviel, established the opinions which had previously been advanced by Maitre Jan. The true nature and seat of the disease now became gradually known. Many fanciful speculations which still existed, have been gradually driven from the schools, and, by the ingenuity of various men of talent and industry, great and important improvements have, from time to time, been suggested on the instruments which are necessary in the operation. Mr. Guthrie presents us with a copious epitome of the opinions of the present day, and we are inclined to regard his personal illustrations with all the attention to which his ability and extensive practice so justly entitle him. He commences his observations upon cataract, by an enumeration of the many distinctions which have been admitted, some of which are as fanciful as they are useless, from their not being available in practice.

"There is, however, a very important distinction, which is frequently neglected, between idiopathic or constitutional, and local or accidental cataracts; not, as in the previous case, referring to the change which has taken place in the structure or appearance of the lens, or to the manner of operating, but to the comfort and happiness of the sufferer. The idiopathic or constitutional disease, affects, in general, both eyes, the local or accidental being more often confined, under proper management, to the organ which has been injured either by external violence or active inflammation." 190.

To prevent the constitutional form of cataract is not in our power. We cannot foresee its commencement. Persons of all ages are subject to it; more particularly, however, those at an advanced period of life. No peculiar temperament enjoys an exemption from cataract, nor has it been found to prevail in those of any known constitution, disposition, or idiosyncrasy. Mr. Guthrie believes that neither scrofula nor syphilis have any share in the production of the disease, unless under particular circumstances, when it is no longer idiopathic. Strumous affections of the eye rarely extend to the interior of the organ. "Syphilis affects the iris with an inflammation, conceived by some persons to be peculiar to the disease, and by contiguity causes an adhesion to the capsule of the lens, producing opacity; but this is an immediate derangement of the part, the effect of which we can predict, and which occurs alike from inflammation not presumed to possess any specific character." However frequent and severe may have been the attacks of syphilis, no facts have yet been adduced to prove that the subjects of them have been particularly liable to cataract. Mr. Guthrie is also opposed to the opinion, that rheumatism and gout have some influence in the formation of the disease. That an hereditary predisposition for cataract, as well as amaurosis occasionally exists, is admitted as a fact for which we cannot account.

"The influence of the constitution in the formation of idiopathic cataract is beyond our means of detection; we can only acknowledge the fact of such an influence existing, and observe the effect, which is in general not confined to one, but extending to both eyes, and with such regularity, that few people who suffer from this complaint in one eye, escape, after a time, the disease in the other; the period between it, in both, is various; occasionally it commences at the same time, although in general one eye is first affected, the other subsequently following the same course; more rarely the second one becomes affected after a considerable interval; whilst few persons live to an extreme old age, having had a cataract in one eye, without having it also in the other. The prognosis, then, in constitutional cataract, as to the probability of one eye escaping, the other being affected, is extremely unfavourable." 193.

The symptoms and appearances common to the various kinds of cataracts are thus described by our author.

"The general symptoms of cataract, both in the idiopathic and accidental diseases, resemble each other at one period of the complaint, yet are of course essentially different in the first instance. In the accidental formation of cataract they are well marked and defined, as being principally *external* or *visible* to our senses. In the idiopathic cataract they are at the same period of formation, and for a considerable time afterwards, for the most part *internal* or *occult*, being invisible to observers and dependent upon the patient's own perceptions, until such time as a change in the structure and transparency of the lens or its capsule has taken place, which may be discovered on a careful inspection.

"When persons are about to suffer from idiopathic cataract, they generally complain of a little weakness of sight, which renders them unable to see objects at as great a distance as formerly; this in a short time increases, so as to render near ones more confused, a greater degree of attention is required to fix and distinguish them accurately, and even then they appear through a mist, or as if seen through a transparent yet turbid fluid, or a glass which has been breathed upon. This indistinctness of vision is constant; no change or rubbing of the eye or motion of the head gives relief, while the patient remains exposed to the same degree of light; but on darkening the room the sight is in some cases, and especially at an early stage, considerably improved, although there is still a central cloud or smoke which cannot be overcome. This advantage is gradually lost in a moderate light, and the patient finds that he is obliged to bring every object nearer his eye to distinguish it correctly, whilst it is often more readily seen from one side than when placed in the axis of vision: he sees best in twilight, when the pupil is most dilated, especially if the opacity be central; and, for the same reason, vision is improved by turning the back to the light; or, in the early stages of the disease, by using clouded glasses, which act in the same manner. On looking at a lighted candle, the flame does not appear as clear and distinct as usual, but seems as seen through a mist and surrounded by a halo, or burr as it is sometimes termed in the North of England; which, when the disease is not complicated with an affection of the retina, is always white or clouded white, and not of various colours, especially either red or blue, or intermixed with what the patient terms flashes of light. This white halo becomes also broader, and the object more indistinct, as it is further removed from the eye. These defects increase with greater or less rapidity, the form of objects is lost, their shadow can, however, be observed between the eye and the light, and the patient is at last only able to distinguish between light and utter darkness, or he may be entirely deprived of every sensation, without having suffered any pain. These essential internal signs are frequently accompanied by others which are not so diagnostic, and which more often appertain to other diseases or derangements. There is frequently an appearance of black specks, of dust, flies, or cobwebs floating before the eyes; flashes of light sometimes dart across them; the candle seems surrounded by burning circles or rings, of divers colours; or a dull pain is felt above the orbit, or at the bottom of the eye; but these symptoms are rather dependent on derangements of the retina or other parts of the eye than of the lens itself; they are not essential to cataract, are with dulness of vision incidental to many complaints, and indicate, when accompanying cataract, the existence of other disease.

"The essential external signs of cataract are infinitely more decisive, inasmuch as many of them are peculiar. In constitutional cataract they may, I believe, be said invariably to follow the internal ones, and the indistinctness and dulness of vision have existed some time before an alteration of structure can be detected in the eye, rendering the diagnosis difficult in many cases, at an early period, between cataract and the mild incipient amaurosis. In a short time, however, the change becomes apparent on a careful examination, a slight general haziness or muddiness may be discovered, which is often of a deeper shade towards the centre in the situation of the lens, giving the back part of the pupil an appearance as if seen through an opaque or muddy substance. The iris is sometimes slightly dilated in a moderate light, compared with the healthy eye of another person; the pupillary edge, especially where the disease is a soft cataract, puts on a darker appearance, as if it were surrounded by a narrow black ring, which is in fact the posterior edge of it, pushed forward in consequence of the increasing size of the lens. If the dilatation be increased to its full extent, by the application of the extract of belladonna, an internal blacker circle will be seen to surround the turbid or muddy part behind the iris, and the patient sees better for a short time than he did previously to its application. The partial loss of transparency soon increases to a state of opacity, proceeding, for the most part in the same manner, from the centre progressively to the edge of the lens; the jet black colour of the pupil is lost, and the space behind the iris is occupied by an opaque body of various shades of colour, from grey to silvery or dead white, to yellow, brown, or a shade approaching to dirty black." 197.

To be correct in our diagnosis and prognosis, particular attention is demanded to the state of the iris, which often distinguishes the constitutional from the local cataract, and marks the complications of disease which may render an operation unadvisable, useless, or improper. Let it not be forgotten that

"The association or sympathy between the eyes is so strong, that the motions of the iris of one eye involuntarily follow those of the other; and if we examine the affected eye, whilst the healthy one is exposed to the same light, we shall have, in all probability, an action of the iris sympathetic with that of the healthy eye, rather than dependent on its own susceptibility for stimuli; whence the rule of covering the sound eye under any examination or performance of operation on the diseased." 198.

Mr. Guthrie makes some important observations upon the different ways in which the motions of the iris are influenced. Our limits oblige us to condense the detailed remarks he offers upon this part of the subject. A healthy state of the iris may *generally* be considered a good, although not an unerring index of the healthy state of the retina. A diseased state, or loss of function of the iris, by no means indicates, although it may lead to a suspicion of a diseased state of the retina. A case is mentioned in which the author operated for artificial pupil on the right eye of a man, who had been blind twenty-two years; no light being transmitted to the retina, and yet at the end of that time it retained its susceptibility for impression, and the patient can now see to read very well.

"It is on the integrity of the healthy susceptibility of the iris for light, and not of the retina, that the contraction and dilatation of the pupil depend in cases of cataract; that a patient suffering from incipient cataract sees best towards evening, or in a moderate light, in consequence of the dilatation of the pupil allowing the rays of light to pass on to the retina through the edge of the lens, which is not so opaque as the centre, and is exposed by the enlargement of the pupil. It is in consequence of the light passing through this edge obliquely, that persons suffering from cataract often see better when they place objects to one side. We endeavour to produce this effect by the application of the belladonna, which causes the pupil to dilate, and allows the transmission of light in the same manner to the retina, which, in general, remains unaffected by it; for, if it were equally under the influence of the application, the patient would not see although light fell on the retina. I have met with several instances of persons using belladonna for months with evident advantage; but still it is a fact equally deserving attention, that in some instances the belladonna seems from the first to paralyze the retina as well as the iris, the sight being either destroyed or rendered much more indistinct until the effect of it had ceased. When given internally and in large doses, it not only influences the motions of the iris, the ciliary and the optic nerves, rendering vision very indistinct, or even destroying sight, but all the nerves of the face connected with the organ of vision; whence its efficacy in some cases of tic doulourex." 201.

By many writers it has been asserted that, if the iris retains its power of motion, the retina is endued with sensibility. It is now known that this rule is liable to many exceptions, and that he who invariably confides in it, will consequently be sometimes led to form an erroneous judgment. In consequence of adhesions being formed between the iris and the capsule of the lens, the motions of the former may be prevented, and it may remain fixed and dilated, or fixed and contracted; or it may be fixed and dilated in consequence of pressure from the lens and parts behind. In such cases the diagnosis is important, and may be accurately formed by suitable attention. If the iris is insensible from deficiency of susceptibility for light, whether natural or acquired by sympathy with the retina, the space between the capsule of the lens and the posterior part of the iris, which is called the posterior chamber of the aqueous humour, is preserved.

"The distance between the edge of the pupil and the lens is perceived, and appears to be more or less natural according to the state of dilatation, which must be taken into consideration. If the belladonna be applied, the pupil becomes fully dilated and the capsule exposed in nearly all its extent; it is often long in returning to its natural state, and it is even possible the pupil may remain partially dilated, but always preserving its circular form. If the immobility of the pupil depend on adhesion between the capsule of the lens and the uvea or posterior part of the iris, it may be suspected from the diminution of the natural space between these parts, by the irregular appearance of the edge of the iris, and by that of the capsule of the lens; it will be proved, and the attachments shown, by forcibly dilating the pupil by the application of belladonna. Immobility of the iris, and especially in the contracted state, is generally the consequence of previous inflammation; it is therefore a local disease, and implies nothing with relation to the retina beyond what we may calculate upon as the effect of inflammation. The iris is generally irregular and puckered, there is no space between it and the capsule; the one adheres to the other; the capsule is always opaque and white, although in a greater or less degree of intensity; and on the continued application of the belladonna, the iris becomes more irregular, if it yield at all, and then shows more marked points of attachment. If the iris be not contracted, or even if it be rather dilated, it always shows the edge of the pupil of a darker colour; and, on the application of the belladonna, it dilates and becomes irregular at the points of attachment, which are then conspicuous. In cases of cataract of long standing, it is possible that adhesions may be formed between the capsule of the lens and the iris, from the simple increase of the lens causing it to press against the iris, or from slight irritation; but in these cases the pupillary edge will seldom or never preserve its natural colour, although it may remain unaltered in shape, whilst the iris may be very sluggish or nearly motionless; but the fact of attachment may be readily proved by the application of the belladonna, and a surgeon is highly reprehensible who does not dilate the iris by it some time before he operates, even if he be certain there is no attachment, because it is the only way in which he can acquire a full view of the surface of the cataract, and in many cases obtain information as to its nature." 203.

Immobility of the iris is not a sign in cataract of paralysis of the retina, especially where there are attachments to the capsule of the lens.

"But where the space of the posterior chamber is entire, or the iris does not seem to be dilated by the pressure of the lens, it is a most unfavourable symptom, and when combined with a total impossibility of distinguishing the shadows of bodies, or of light from darkness, it nearly amounts to a prohibition of the operation, which ought on no account to be performed, if it be accompanied by pain in the eye, orbit, or forehead, or with flashes of light shooting through the eye, which indicate amaurosis, or approaching disorganization. When the immobility of the iris only marks its want of sensibility, and the patient can readily distinguish light from darkness, there is good authority for performing the operation, and it is right to attempt it, where the patient has suddenly been deprived of that power from inflammation, because it may have occurred in consequence of adhesions. But if it have suddenly occurred, the pupil remaining of its natural size, or being dilated, there is little or no hope of success, and particularly if both eyes are affected." 204.

To prove the necessity of closing the sound eye during the examination of the diseased one, Mr. Guthrie mentions an interesting case in which the patient "is completely blind, so as to be incapable of distinguishing light from darkness. The iris is slightly discoloured, the pupil is black and of a moderate size, and remains unaffected by the stimulus of light when allowed to fall upon it alone, although it is enlarged or diminished according to the motions of the iris of the opposite eye. When the sound eye is covered, the pupil of the diseased one is immediately dilated to a moderate extent, and remains in that state, and immoveable even under the influence of the full glare of the sun. But if the sound eye be uncovered and exposed to the same degree of light, both pupils are instantly contracted. If a cataract were to be formed in this case, the state of the iris might escape detection, unless carefully inquired into when the sound eye was closed." This sympathy of the iris of one eye with that of the other, when the sympathy between the iris and retina of the same eye is destroyed, is not unfrequent, and should be constantly borne in mind.

"The iris must also be examined as to the correctness of the manner in which its motions of contraction and dilatation are performed. In the natural state of the eye, the iris is a perfect plane not protruding forwards or slanting backwards, and is without folds or plaits. A deviation from this appearance indicates derangement, in consequence of pressure from behind, or of its own structure, either of which may influence the mode of operating, as will be hereafter mentioned. The plane of the iris is preserved by a due degree of pressure before and behind, anteriorly by the aqueous humour supported by the cornea, and posteriorly by the quantity of the aqueous humour contained in the posterior chamber supported by the peculiar firmness of the crystalline lens. In this space the iris moves, displacing from one part to another the aqueous humour as it either dilates or contracts; but this is accomplished with such precision, and the balance of pressure appears to be so little disturbed, that the iris performs these motions without any vacillation apparent to our sight, a fact of great importance as a diagnostic sign of a lenticular cataract; for it appears that it is mainly on the presence and consistency of the lens that the equilibrium of pressure depends, and not on the fulness of the eye, for the increase of aqueous, or of vitreous humour, the lens being removed, or even dissolved in its proper capsule, will not answer the purpose, the iris obtaining under these circumstances a tremulous motion, indicating the absence of the lens. This circumstance is frequently attributed to dissolution of the vitreous humour, which is also capable of giving rise to it; but I am satisfied, it is not the most common cause in cases of cataract; for, in all those on which I have operated with restoration of sight, when this sign was present, I have found the cataracts to be capsular, the lens having been absorbed or become fluid. In these cases previous pressure on the eye with the point of the finger had convinced me, by the resistance it met with, that the vitreous humour was of a proper consistence; and in two cases in which I operated by an opening in the cornea, a sufficient quantity of the vitreous humour escaped in removing the capsule, to enable me to ascertain the fact and to convince me of the propriety of the opinion I have formed as to the cause of such appearance. In all cases of cataract, then, pressure should be made on the eyeball with the finger, to ascertain the degree of consistency of the vitreous humour; if thin and watery, the eye will yield to it, and the iris will acknowledge the pressure in a very decided manner, when the disease is advanced to the tremulous state I have alluded to, and which is best seen under general motion of the eye exposed to great variations of light and shade." 206.

In a note, Mr. Guthrie observes that the above remarks on the motions and sympathies of the iris have formed a part of his lectures, and have been publicly delivered at the Infirmary twice every winter for the last six years, independently of the necessary recurrence to them on many other occasions, in pointing out the symptoms diagnostic of cataract from other diseases. The Baron Larrey in France, and Mr. Shaw in London, have within the last year published a part of them as peculiar to their observation. Important information is to be gained from an examination of the cataract itself: its situation and influence upon the iris having been previously estimated the oculist should endeavour to ascertain the changes of structure or quality which have taken place, from the appearances the part may have assumed. This discrimination is confessedly difficult. The great Scarpa is of opinion that it is not easy to pronounce whether the cataract be hard or soft, caseous or fluid, and whether, together with the opacity of the crystalline lens, the capsular membrane which envelopes it be also opaque.

"In this opinion," says Mr. Guthrie, "Scarpa is supported by all the oculists in Europe, with the exception of Sir William Adams," who conceives the learned Professor is mistaken upon this point. Sir William admits that no verbal or written description can convey to an inexperienced practitioner an accurate idea of the various kinds and shades of cataract, but contends that an oculist of just and accurate observation will rarely be deceived in his opinion upon the nature of the cataract upon which he is to operate.

We must refer to the work itself for the diagnosis between cataract and other diseases. The causes of cataract are still enveloped in considerable obscurity, "which is not likely to be cleared away, until the nature and formation of the lens is better understood." In the opinion of Mr. Guthrie, many of the exciting causes which Professor Beer enumerates are referrible to amaurosis, and many of them may be doubted as applicable to cataract.

The following observation of our author demands attention.

"It is a fact which I wish to impress on the minds of students, that those inflammations of the iris and ciliary processes, which are active in their nature, and quickly cause a deposition of lymph, and in considerable quantity, are the most easily recovered, and the capsule of the lens thereby restored to its natural state, unless the disease has been too long neglected; whilst those inflammations of the same parts which are slow in their progress, and cause the deposition of a small quantity only of lymph scarcely perceptible, but from its effects in attaching the iris to the capsule of the lens, are very difficult of cure, and generally leave some defect. In the former cases, when neglected, the disease terminates in cataract, with an adherent iris, and possibly a closed pupil. In the latter the pupil becomes irregular, is attached to the capsule of the lens, which becomes opaque in points, and may be completely so. The eye is likely to become amaurotic, or glaucomatous, from repeated recurrences of the inflammation, if that which has already taken place has not been sufficient for the destruction of vision." 227.

Various classifications of cataract have been adopted by various authors. Some arrangement is necessary; and Mr. Guthrie properly selects that which is the most efficient, and least troublesome. He divides cataracts into two classes. The true, and the false, or spurious cataracts.

"The first class, or the true cataracts, containing three genera, or all those, of whatsoever description they may be, which have been caused by derangement or disease of the lens or its capsule, or by both; but unconnected with any perceptible derangement or attachment to the iris or adjacent part.

"Three genera.

"1. Cataracta lenticularis, or lenticular cataract.

"2. Cataracta capsularis, or capsular cataract.

"3. Cataracta capsulo-lenticularis, or capsulo-lenticular cataract.

"The second class, or the false cataracts, containing all those previously noticed, or otherwise, which are combined or connected with derangement of the iris, or adjacent parts, as a consequence resulting from inflammation.

"The term *complicated* may be retained to mark the presence of other and more important diseases, such as amaurosis, glaucoma, cirsophthalmia, &c." 227.

The lenticular cataract is divided into four species. 1. The hard. 2. The fluid. 3. The soft. 4. The caseous. Several pages are occupied upon the distinguishing symptoms of these various kinds of cataract, which, as we have stated already, require a well-practised eye for their detection. Upon the subject of spurious cataract, it is to be observed that,

"The history of a case of false cataract is very important, as it will frequently point out its nature, and with the appearances of the part itself, indicate what probability there is of success attending an operation for its removal. A false cataract being the consequence of injury or of inflammation, its formation has been attended by more or less pain, which is frequently violent, and often followed by extinction of sight. In all such cases, the iris, the choroid coat, and the retina, will have become diseased, and it may be readily complicated with amaurosis, glaucoma, or cirsophthalmia, the symptoms of which will be, in general, sufficiently evident. Those which are deduced in amaurosis from the state of the pupil, and the susceptibility for light must be defective, inasmuch as the iris is attached to the capsule of the lens, which is itself thickened, and nearly impermeable to light. Shadows of objects passing between the eye and the sun, on a clear day, can be observed in most persons whose eyes are not amaurotic. The pupil, in the majority of these cases, is diminished in size, the edge of it firmly attached to the capsule of the lens, and although a part of it is visible, the opening would be too small for correct vision, even if the opacity could be removed. They come, therefore, under the consideration of those states of the eye which require an operation for *closed* or for an *artificial pupil.*" 245.

*On the Cure or Removal of a Cataract.* Mr. Guthrie is by no means sanguine in his expectations of curing even incipient cataract by medicinal means. Opacities of thecapsule of the lens may be sometimes arrested, or even removed, when they arise as the consequence of common inflammation, and do not constitute the true idiopathic capsular cataract. To determine the proper period for operating is evidently of importance. The better the general health of the patient, the greater is the probability of success. In some cases, previous preparation may be necessary, whilst in others it will not be required. The patient being temperate, and in good health, will need only to abstain from animal food for a couple of days before the operation, and to take an aperient. The operation should, if possible, be avoided during pregnancy. Much contrariety of opinion exists as to the propriety of operating upon one eye whilst the other remains sound. Mr. Guthrie is decidedly of opinion that, as far as the improvement of sight is concerned, the operation should not be attempted on one eye, if the other is in a healthy state. It is not possible to determine positively how far the removal of the cataract in one eye may delay or prevent the formation of it in the other. The performance of the operation upon both eyes at the same time, has, by some authors, been strongly recommended, and by others, as urgently deprecated. Scarpa* recommends us to wait till the eye first operated on is well. Beer adopts a middle course; he is of opinion that, when cataracts are completely formed, and every thing promises a favourable result, both eyes may be operated on at the same time. In this opinion Mr. Guthrie concurs; he adds "that if any accidental circumstance should take place during the first operation, rendering its success doubtful, the second should be delayed to a future opportunity."

*The operations* for cataract have, from time to time, undergone many changes, both in the mode of performing them, and in the instruments by which they are accomplished. It would be to no purpose to detail the many progressive improvements

* Scarpa, by Briggs, page 356.

which have been suggested by the gradual detection of erroneous principles.

*On the Operation by Displacement.* Mr. Guthrie observes that this operation has been usually termed couching or depression, which was sufficiently comprehensive so long as there was but one mode of performing it. Since several methods have been recommended, not only differing essentially from each other in the operative process, but in the manner of displacement, and the subsequent position of the lens, it is advisable to have a term for the operation generally, which may not interfere with, and lead to a false conception of each kind of operation individually. The operation by displacement, is divided by our author, First, into operations posterior to the iris; containing 1, simple depression; 2, depression of Scarpa; 3, reclination of Willberg and Beer. Secondly, into operations anterior to the iris. Reclination through the cornea.

*On the Operations posterior to the Iris.* 1st. Simple depression, consisting in dislodging the opaque lens from its natural situation, and placing it in the vitreous humour, so far from, and under the level of the pupil that vision may not be prevented.

"It is fairly divisible into three parts. 1. Introduction of the needle. 2. Placing it on the anterior surface of the lens. 3. Removing the lens out of and below the axis of vision."

"The patient is to be placed, and the eye fixed in the manner recommended for extraction, the pupil having been previously dilated by the belladonna. The small spear-pointed needle is to be held steadily but lightly, like a writing pen; the little finger resting on the cheek bone, or side of the orbit, so as to give due support to the hand, and prevent the needle from entering violently, or going too far, if the toughness of the sclerotica should require a considerable degree of pressure to be made. It should penetrate the sclerotica about a line and a half from the edge of the cornea (not nearer than a line, not farther than one and a half) and half a line below its horizontal diameter, to avoid the long ciliary artery; the point being directed towards the centre of the eye,* so that it does not enter directly on the lens, and change its position, pushing it towards the nose; one flat surface of the needle should be upwards, the other downwards.† The triangular point of the needle is to penetrate in this direction, until the neck of the instrument has entered the wound. The first step of the operation is then completed, and the second begins with a double motion of the needle, which requires a little dexterity. The direction of the point of the needle is to be changed from the centre of the eye towards the nasal edge of the pupil, which can only be done by carrying the handle backwards towards the temple, and as it approaches this new direction, it is to be turned so far (a quarter of a circle

---

* Hey's Surgery, page 63. Beer, Leitfaden, s. 88.

† Warner, page 95.

forwards) on its axis, that the flat surface, which at the commencement of the operation was upwards, may now be turned towards the operator. It is in fact in the position it would be in, if it had been introduced in the manner more commonly adopted, and it is to be passed on in its new direction, until its point and flat surface are seen advancing behind the temporal edge of the pupil. It is to be carried on in the posterior chamber, between the iris and the cataract, until the point has fairly passed the nasal edge of the pupil. One flat surface is towards the cornea, the other pressing on the cataract. The second stage is now completed.

"These two stages are equally applicable to the operation of reclination through the sclerotica, and the knife is in the same situation as it would be in for the division or breaking up of a soft cataract, save that it is not quite so far across the lens towards the opposite side.

"The third stage commences by a double motion, resembling that of the second; the handle of the instrument is to be depressed, so that the point of the needle may be elevated to the upper edge of the cataract, on which the broad flat surface of the instrument must be placed, by giving it a quarter turn backwards on its axis. The posterior flat face of the needle, which pressed against the face of the cataract, is now on its upper edge, and the needle is (excepting the elevation of its point) in the same situation as when it was first introduced. The third stage is now completed by raising the handle, and firmly depressing the cataract downwards, and a little outwards. The handle of the needle should not be raised above the horizontal position, but it should be kept there steadily for a few seconds, to prevent the lens rising again, when the point is to be gently raised. If the lens remains depressed, the object of the operation is completed, and the needle is to be withdrawn in the same manner as it was introduced, only with the motions in an inverse order. If the lens rises up after the instrument, the depression must be repeated, and again a third or fourth time, with a longer interval between each attempt, until it can be lodged below the level of the pupil." 269.

For the performance of the operation, Mr. Guthrie prefers Beer's needle. He considers it a point of great importance, and fully established in this operation, that the lens is on no account to be pierced, and that the integrity of the lens is to be preserved, if necessary, at the expense of the ciliary processes. In opposition to Beer, and the oculists of the German school, Mr. Guthrie maintains that either the lens or the ciliary processes will be injured. He considers the dangers which have been said to arise from division of the ciliary processes to be much exaggerated, and remarks,

"That the ciliary processes are parts of great importance, in a healthy state of the eye cannot be doubted, from an examination of their structure and distribution; but that a principal part of their utility terminates with the removal of the lens, is, I think, almost equally well demonstrated in many cases of operation, especially by division, where they must have sustained considerable injury, and yet the vision of the patient has been as good, and continued so, as in any other case of cataract after operation. I have seen this very often, I have done it repeatedly, and therefore affirm it as a fact; but it is not a lesion of the functions of the ciliary processes that is, we are told, principally to be dreaded from wounding them, but instant hæmorrhage into the chambers of the eye, obscuring the view of the parts through the pupil, preventing the completion of the operation, or rendering it necessary to finish it as it were in the dark, and incurring the risk of a great and destructive inflammation. If this were actually a fact, I am not certain which of the two injuries would be the worst; but I have no hesitation in saying that these dangers are imaginary, not real, that the ciliary processes may almost always be cut or divided without fear of their bleeding; I have cut them repeatedly, without this or any bad effects whatsoever, either at the moment or any subsequent time." 272.

The operations of Scarpa, Willberg, and Beer, are given at full length. We must refer to the work for their description, and for many ingenious observations suggested by Mr. Guthrie upon the subject. He is of opinion that the choice of the operation, in able hands, is nearly a matter of indifference. He prefers, however, the operation of reclination.

The operation of reclination through the cornea, by introducing the needle anterior to the iris, has been principally introduced and supported by Professor Langenbeck of Gottingen, and is intended for hard cataracts only.

*After-treatment.* The operation having been completed, examinations to ascertain whether the patient can see are useless and improper. "As the needle is withdrawn, the eye-lid ought to be allowed to drop, and need not be re-opened." The admission of light is to be prevented, a suitable bandage applied, and low diet observed for several days. On the third or fourth day, the eye may be opened, with the back turned to the light. It will be found more or less inflamed, and must be treated according to circumstances. Violent vomiting sometimes comes on immediately, or a few hours after the operation. At any period this occurrence is unfortunate, as it may, from the exertion and shock, cause the lens to reascend, and in addition to the injury already committed, prove a source of greater mischief, by irritating the iris. Opium, saline draughts, and camphor should be given to allay this distressing symptom. Upon the subject of the operation by extraction, a considerable space is occupied. The opinions of various authors are stated, and the mode of operation minutely described. Considerable dexterity and experience are necessary in the performance of it. For a mass of important information upon the subject, we must refer our readers to the work itself,

*On the Operations for the Division, or Breaking-up of the Cataract.* 1. Posterior to the iris. 2. Anterior to the iris, or through the cornea. The keratonyxis. Mr. Saunders tried several methods of proceeding, both posterior and anterior to the iris; first, by breaking up the lens, and pushing it into the anterior chamber of the aqueous humour, and latterly, by merely opening the centre of the capsule, and allowing the lens to remain in situ until removed by the absorbent process. "Further experience," says Mr. Guthrie "has not decided in favour of the latter method, and I consider the former to be the better operation, as facilitated by the use of the two-edged needle, recommended by Sir William Adams, whose method of proceeding is described in his work.* The operation for dividing or breaking up a cataract, should never be attempted when the lens is hard, according to the opinion of Mr. Guthrie.

The operation anterior to the iris, is not recommended by the author as one to be preferred for the removal of a cataract, although, he observes, "I have found it useful as a preliminary step, where the cataract has been very soft and large, pressing against the iris, and rendering it convex."

*The Operation for Capsular Cataract.* An operation for capsular cataract alone necessarily implies the previous removal of the lens, either by absorption or operation. The removal of the capsule from the axis of vision is difficult of accomplishment, and sometimes several operations are necessary before the desired success is obtained. Mr. Guthrie recommends,

"That the capsule be first separated as much as can be conveniently done, from its attachments, by one operation posterior to the iris, with the needle, and then extracted at a subseqent period, through a small opening in the cornea; for a repetition of the use of the needle will be attended by the same difficulty, and at last recourse must be had to evulsion and removal through the cornea. The eye being fixed, Scarpa's needle, or one (which I prefer) less bent, being slightly curved towards the point, and cutting on both edges, is to be introduced and brought in front of the capsule in the usual manner. It is then to be carried to the opposite side, the pupil having been previously dilated by the belladonna as much as possible, and the capsule is to be separated from its attachments: by working with the edges and points of the needle, it will give way in parts, under gentle pressure; but other points of attachment of great firmness will remain, appearing to resist almost any degree of pressure that can be applied by either the cutting edge or point of the needle. As much, however, as can be done consistent with the safety of the eye under such circumstances, is to be attempted for the purpose of loosening or separating the capsule; and if this should be happily effected, it may be pushed into, if it cannot be thrust through, the pupil. The inflammation which may ensue having subsided, the second operation is to be performed in a few days, in the following manner: the patient must be placed on his back, and the external part of the cornea opened for near one fourth of its extent, but where the transparent cornea is large, one fifth will be sufficient; for, if room be not given for the easy introduction of the instruments, the irritation in passing them in will be the cause of a greater subsequent opacity of the cornea, than the mere

* Sir William Adams's New Operations for the Cataract, p. 225.

size of the incision can possibly be, whilst the chance of a protrusion of the vitreous humour will be rather diminished than increased; for, if the opening be sufficiently free, the flap rises, and there is no pressure on the ball of the eye; but if the opening be confined, it is the sclerotica that yields, and the vitreous humour is compressed. Two instruments ought now to be at hand, a small but sharp hook, and a pair of spring forceps serrated within the points. I generally first empoly the hook, by passing it into the pupil, and under the capsule, which being pierced upwards, is to be drawn steadily but not forcibly out of the eye; sometimes it will yield, and the operation is almost immediately completed; at others it may be drawn just without the cornea, and its attachment divided with the scissors, or it may be so tough that the hook will not take sufficient hold, and slip, or bring away only a piece. I then try the forceps, which are to be introduced shut until they reach the capsule, when the blades are to be opened, and made to close on as much as possible of the membrane intended to be removed; the spring will now keep the blades together, and prevent the capsule slipping from the points of the forceps, which are serrated within. If the forceps be now drawn out, it is evident the capsule must come with them; but in doing this, the surgeon will sometimes perceive that he turns the hyaloid membrane on its axis, or that he pulls it so much towards him, that the vitreous humour is compressed against the side of the sclerotica, and bursts from its cells, a portion being evacuated; for the hyaloid membrane, in many cases of this kind, becomes exceedingly dense and strong, much beyond what might be conceived from an examination of its healthy structure. The capsule should not then be forcibly torn out, but the forceps turned on its axis, by which means the capsule is wound round the blades of it, the evulsive force is more equally divided on the surface of the hyaloid membrane, and is more easily regulated. If this operation fail, the scissors must be introduced, and the attachment divided as close as circumstances will admit. Proceeding in this way, and with due caution, greater liberties may be taken with the eye than could be supposed, and with perfect safety; for the inflammation, if any follow, is very manageable by simple means." 339.

For a description of the compound operation of displacement and extraction, we must refer to the various authors who have considered the subject, or to Mr. Guthrie's book, which contains an abstract of their opinions. Mr. Guthrie concludes the subject of cataract with a detailed consideration of the advantages and disadvantages attending the various modes of operating, as applicable to the different species of cataract. This subject has undergone frequent and warm discussion, and oculists are still far from being agreed as to the particular operation demanded for each peculiar case. The volume terminates with a chapter on artificial pupil. Thus then, we have at length arrived at the termination of Mr. Guthrie's work, and we candidly confess we have seldom had a more difficult task presented to us than that of offering to our readers an abstract of this volume, without trespassing very much beyond the limits to which we are sometimes unwillingly compelled to restrict ourselves. They who are interested in the operative surgery of the eye, will here find a mass of important information. The opinions of most authors who have written upon the subject are candidly stated, and cannot have been collected without immense labour. Every author who professes to give a systematic work upon so extensive a subject as the operative surgery of the eye, must necessarily state much that has before been stated. "*Vera non nova*" must be his chief maxim. For this additional proof of talent and unwearied zeal Mr. Guthrie not only deserves, but will receive the thanks of his professional brethren.

Mr. Stevenson's work now comes under our notice. The object of it is to make known the more mature fruits of Mr. S's experience, since the appearance of his last production on cataract. The intention is laudable; the mode of its execution is now to be examined. In the last page of the preface, we are informed, that "the omission of a longer list of cases has arisen, not from any deficiency in their number, but because the author deemed it superfluous on the one hand to record them, and on the other, their insertion would tend to augment the size and enhance the price, rather than add to the value, of the work." We augured well from this sentiment, and sat down to the perusal of the book with an expectation of at once being introduced to "a mode of practice calculated to prevent the occurrence of blindness, and to render unnecessary the operations of couching and extraction." A reviewer, however, is not unfrequently doomed to wade through some pages of common introductory matters, the insertion of which "tends to augment the size and enhance the price, rather than add to the value of the work." We will not inflict upon our readers that of which we ourselves complain. We shall pass over, the well known and oft repeated particulars of the practice of "Galen, the cotemporary of Antoninus Pius, the 16th Roman Emperor," the chapter on the different species of cataract, and that on the removal of cataract by couching and extraction, with one single observation on the latter. It appears to us that the difficulties and dangers of couching and extraction, are not so great in the hands of a skilful operator, as they are represented by Mr. Stevenson. Neither do we admit that the number of cases to which any one of the several processes of the operation by displacement is applicable, "is very limited." Cases do occur in which the usual operations are not applicable, but they are very rare, comparatively, to those in which success may be obtained.—The 4th chapter "on the advantages resulting from the removal of the different species of cataract, at an early period after their formation, by the absorbent practice" merits attention. Mr. Stevenson observes, that however "dissimilar the mode of performing the several operations, they have all been reserved for that stage of the ailment, when the sight has become greatly impaired or wholly destroyed." He, on the contrary, recommends the removal of the cataract as "soon as its character is sufficiently disclosed to enable us to decide upon the real nature and tendency of the disease." The main object in conducting the mode of treatment termed the absorbent practice "is to comminute with a proper needle, the opaque crystalline, or tear its capsule, when it is morbidly affected, into small shreds or filaments, in order that they may in these states more readily undergo the process of solution in the aqueous humour, or be acted upon by the absorbents, whilst, at the same time, the smallness of their size tends to obviate pressure and consequent irritation of the adjacent parts." In other words, the patient is to be couched *at an early period of the disease*, and by this mode of proceeding, whatever disadvantages might arise from allowing the cataract to remain unmolested for an indefinite time will of course be obviated. The suggestion is valuable. To confirm the superiority of the plan recommended, Mr. S. observes "if the proposed operation be undertaken at the *early* period I have so strongly recommended, and *before* any central nucleus has formed, I am enabled to speak from reiterated and far more extensive experience in these cases than has fallen to the lot of any other person (who being unacquainted with my views on the subject, has never adopted the practice inculcated in these pages) that inflammation is so *rare* a contingency when my needle is used with the requisite delicacy and address, or in the event of its taking place is so easily removed, as to give no cause for uneasiness or apprehension. In confirmation of this fact, were it not deemed superfluous, I could produce cases, in number, adequate to fill a volume." If the first 138 pages of Mr. Stevenson's book, had been omitted, the contents of which are to be found in many other authors, and a part only of these cases had been given, to us, and to the profession at large, the work would have been more satisfactory, and its object more fully achieved. Mr. Stevenson's experience has convinced him "that by operating at an *early* period after the formation of cataract in one eye, the *commencing* disease in the other has, in some instances been immediately arrested; whilst, in several, the organ, in which the opacity had made greater progress, so perfectly regained its former pellucidity and healthy function, as to render an operation in that eye altogether unnecessary."

Mr. Stevenson we presume is aware of the advantages of an imposing title page. Judgment is not unfrequently passed both upon books and men without a perusal of the interior. The contents of a title page however are sometimes as deceptive as the features. "To render unnecessary the operations of couching and extraction" is the principle intention which strikes the eye in the title page of Mr. S's book. The sum and substance of the work itself, however, is simply to *recommend* the operation of couching at an early period of the disease.

It is true the lens is not *depressed*, but when the cataract is punctured and disturbed in the manner proposed by Mr. Stevenson, the term couching is extended to the operation. Mr. Saunders performed it with success upon many children, and oculists are frequently satisfied by the mere disturbance and comminution of the lens. We read every production of Mr. Stevenson's with a *disposition* to commend. The volume before us might furnish sufficient matter to constitute an interesting article in a Medical Journal, but some ingenuity has been required to stretch it into a book of 234 pages.

The object of Dr. Bowen's book is to recommend an operation which he denominates hyalonyxis, or vitreous operation in every species and variety of cataract.

The *term* hyalonyxis is the property of Dr. Bowen; the operation itself has been long known and practised notwithstanding some of our cotemporaries have awarded the merit of its introduction to him. Dr. Bowen's success has certainly been great. In one hundred and sixty cases performed by him, general bleeding was only required three times, and *no* case of secondary membranous cataract occurred. Dr. Bowen presumes he has first suggested,—

1st. To penetrate the sclerotic far backwards. To prove that this is no novelty, see Sharp's Plates in Surgery, and Mr. Guthrie's observations on the subject.

2d. To dilate the pupil previously to the operation.

This expedient to facilitate the subsequent steps of the operation is well known and generally practised. In a letter written by Scarpa to our friend Mr. Briggs, the indiscriminate application of belladonna previous to operating upon the eye is deprecated. No reason however is assigned for the objection by this able and intelligent surgeon.

3rdly. The manner of destroying the capsule.

A reference to Mr. Guthrie's book, will shew that he has always done it in the same manner. The success of Dr. Bowen is highly creditable to his operative dexterity. To novelty he has no claim.

